# SEPSIS-3.0 – Ist die Intensivmedizin bereit für die ICD-11?

**DOI:** 10.1007/s00101-021-01012-8

**Published:** 2021-08-05

**Authors:** Thomas Schmoch, Michael Bernhard, Andrea Becker-Pennrich, Ludwig Christian Hinske, Josef Briegel, Patrick Möhnle, Thorsten Brenner, Markus A. Weigand, SepNet Study Group

**Affiliations:** 1 Klinik für Anästhesiologie, Universitätsklinikum Heidelberg, Heidelberg, Deutschland; 2 Klinik für Anästhesiologie und Intensivmedizin, Universitätsklinikum Essen, Hufelandstr. 55, 45147 Essen, Deutschland; 3 Zentrale Notaufnahme, Universitätsklinikum Düsseldorf, Heinrich-Heine-Universität, Düsseldorf, Deutschland; 4 Klinik für Anästhesiologie und Abteilung für Transfusionsmedizin, Zelltherapeutika und Hämostaseologie (ATMZH), Ludwig-Maximilians-Universität (LMU) Klinikum München, München, Deutschland; 5 Institut für medizinische Informationsverarbeitung, Biometrie und Epidemiologie, LMU München, München, Deutschland

**Keywords:** Kodierung Sepsis, Sepsis-Definiton, QSOFA, Infektion, Umfrage, Encoding, Sepsis definitions, QSOFA, Infection, Survey

## Abstract

**Hintergrund:**

Im Januar 2022 wird die 11. Revision der International Classification of Diseases (ICD-11) in Kraft treten. Unter anderem wird darin die SEPSIS-3-Definition implementiert sein, in der Sepsis als „lebensbedrohliche Organdysfunktion, hervorgerufen durch eine fehlregulierte Wirtsantwort auf eine Infektion“ definiert wird. Ziel der vorliegenden Sekundärauswertung einer Umfrage zum Thema „Sepsis-induzierte Koagulopathie“ war es zu evaluieren, ob die SEPSIS-3-Definition (engl. The Third International Consensus Definitions for Sepsis and Septic Shock) 4 Jahre nach ihrer internationalen Einführung im klinischen Alltag anästhesiologisch geführter Intensivstationen in Deutschland angekommen ist und so die Voraussetzungen für die Verwendung des ICD-11 gegeben sind.

**Methoden:**

Im Rahmen einer deutschlandweiten Umfrage unter ärztlichen Leitern von Intensivstationen, die zwischen Oktober 2019 und Mai 2020 durchgeführt wurde, wurde in einem gesonderten Fragenblock nach der verwendeten Sepsisdefinition gefragt. Zusätzlich wurde gefragt, ob der quick-Sequential (Sepsis-related) Organ Failure Assessment (qSOFA) Score zum Screening auf Sepsis in dem Krankenhaus, zu dem die teilnehmende Intensivstation gehört, verwendet wird.

**Ergebnisse:**

Insgesamt nahmen 50 ärztliche Leiter von anästhesiologisch geführten Intensivbereichen an der Umfrage teil. In Summe gaben die ausgewerteten Intensivstationen an, etwa 14,0 % der in Deutschland registrierten High-Care-Betten zu führen. An 78,9 % der Universitätsklinika und 84,0 % der teilnehmenden Lehrkrankenhäuser ist die SEPSIS-3-Definition im klinischen Alltag integriert. Im Gegensatz dazu wird der Screening-Test „qSOFA“ nur von 26,3 % der teilnehmenden Universitätsklinika, aber immerhin von 52,0 % der Lehrkrankenhäuser und 80,0 % der „sonstigen“ Krankenhäuser verwendet.

**Schlussfolgerung:**

Unsere Daten zeigen, dass sowohl SEPSIS‑3 als auch qSOFA im klinischen Alltag deutscher Krankenhäuser angekommen sind. Die zurückhaltende Verwendung des qSOFA an Universitätsklinika bei gleichzeitiger breiter Akzeptanz der SEPSIS-3-Definition kann als Indiz interpretiert werden, dass die Suche nach einem geeigneten Screeningtest für Sepsis noch nicht abgeschlossen ist.

## Abstract (en)

**Background:**

The 11th revision of the International Classification of Diseases (ICD-11) will come into effect in January 2022. Among other things, The Third International Consensus Definitions for Sepsis and Septic Shock (SEPSIS‑3 definition) will be implemented in it. This defines sepsis as a “life-threatening organ dysfunction caused by a dysregulated host response to infection”. The aim of the present secondary analysis of a survey on the topic of “sepsis-induced coagulopathy” was to evaluate whether the SEPSIS‑3 definition, 4 years after its international introduction, has arrived in everyday clinical practice of intensive care units (ICU) run by anesthesiologists in Germany and thus the requirements for its use of the ICD-11 are given.

**Methods:**

Between October 2019 and May 2020, we carried out a nationwide survey among German medical directors of ICUs. In a separate block of questions we asked about the definition of sepsis used in daily practice. In addition, we asked whether the quick-sequential (sepsis-related) organ failure assessment (qSOFA) score is used in screening for sepsis in the hospital to which to the participating ICU belongs.

**Results:**

A total of 50 medical directors from anesthesiological ICUs took part in the survey. In total, the ICUs evaluated stated that they had around 14% of the high-care beds registered in Germany. The SEPSIS‑3 definition is integrated into everyday clinical practice at 78.9% of the university hospitals and 84.0% of the participating teaching hospitals. In contrast, the qSOFA screening test is only used by 26.3% of the participating university hospitals, but at least 52% of the teaching hospitals and 80% of the other hospitals.

**Conclusion:**

The data show that both SEPSIS‑3 and qSOFA have become part of everyday clinical practice in German hospitals. The cautious use of qSOFA at university hospitals with simultaneous broad acceptance of the SEPSIS‑3 definition can be interpreted as an indication that the search for a suitable screening test for sepsis has not yet been completed.

## Hintergrund

Die „International Classification of Diseases 11th Revision“ (ICD-11) wurde im Mai 2019 verabschiedet und soll am 01.01.2022 in Kraft treten [10, 22]. Darin implementiert ist auch die Dritte Internationale Consensus Definition für Sepsis und Septischen Schock (SEPSIS‑3-Definition), die seit 2016 gültig ist. Hierin wird Sepsis als „lebensbedrohliche Organdysfunktion, hervorgerufen durch eine fehlregulierte Wirtsantwort auf eine Infektion“ definiert [18]. Ein septischer Schock wird darin als eine „Unterform der Sepsis“ definiert, bei der „die zellulären, metabolischen und kardiozirkulatorischen Veränderungen“ so schwerwiegend sind, dass sie mit einer signifikant höheren Letalität assoziiert sind als bei einer „einfachen Sepsis“ (Letalität der Sepsis ca. 10 % vs. ≥ 40 % bei einem septischen Schock) [18]. Operationalisiert wird eine Organdysfunktion dabei durch einen Anstieg des Sequential Sepsis-related Organ Failure Assessment(SOFA)-Scores um ≥ 2 Punkte und der septische Schock durch das gleichzeitige Vorliegen (1) einer Sepsis mit (2) einem erhöhten Serumlaktatwert von ≥ 2 mmol/l (ca. 18 mg/dl) und (3) einem Katecholaminbedarf trotz einer ausreichenden Flüssigkeitstherapie (in der Regel initial 30 ml/kgKG). Diese Definitionen haben die SEPSIS-2-Definitionen abgelöst, die bei der Sepsis 3 Schweregrade unterschieden: Eine „einfache Sepsis“ war gemäß SEPSIS‑2 definiert als das Zusammenkommen von ≥ 2 Systemic-Inflammation-Response-Syndrom(SIRS)-Kriterien (Liste 1) mit einer (wahrscheinlichen) Infektion. Eine „schwere Sepsis“ wurde durch eine Organdysfunktion im Rahmen eines SIRS charakterisiert und ein „septischer Schock“ ganz allgemein durch eine Hypotension bzw. Katecholaminpflichtigkeit trotz ausreichender Flüssigkeitszufuhr infolge einer Sepsis [13]. Diese Dreistufigkeit ist auch in dem Vorgänger des ICD-11, dem „ICD-10“ abgebildet, der wiederum bis heute die Grundlage für die Kodierung und Vergütung in Deutschland darstellt [21]. Dabei steht der Code R65.0! für eine Sepsis gemäß der alten Definition (2 SIRS-Kriterien und 1 positive Blutkultur oder 4 SIRS-Kriterien ohne Blutkultur) und R65.1! für eine „Sepsis mit Organkomplikation“. Da diese Unterscheidung offensichtlich nicht mehr zweckdienlich ist (da es gemäß SEPSIS‑3 keine Sepsis ohne Organkomplikationen mehr gibt), schlägt die Deutsche Sepsis Gesellschaft (DSG) in Übereinstimmung mit der World Health Organisation (WHO) auf ihrer Webseite vor, den ICD-10 Code *A41***.**– („Sonstige Sepsis“) in Kombination mit einer Kodierung der Organkomplikation(en) zu verwenden [5], um so die Divergenz zwischen Definition und Kodierungsmöglichkeiten bis zum Inkrafttreten des ICD-11 zu überbrücken.

Dieser nicht einfach zu überschauende Übergangszustand könnte dazu beigetragen haben, dass selbst im Jahr 2018 (also 2 Jahre nach der Einführung der SEPSIS-3-Definitionen), nicht einmal die Hälfte der Intensivstationen in Deutschland (43 %) die Veränderungen der Definitionen in ihren Standardarbeitsanweisungen implementiert hatten [11]. Ziel der vorliegenden Umfrage war es zu evaluieren, ob die SEPSIS-3-Definition 4 Jahre nach ihrer internationalen Einführung im klinischen Alltag deutscher Intensivstationen angekommen ist.

***Liste 1: SIRS – Kriterien (mod. nach [*1*])***
- Tachykardie > 90/min
- Tachypnoe > 20/min oder p_a_CO_2_ < 32 mm Hg
- Hyperthermie > 38 °C oder Hypothermie < 36 °C
- Leukozytenzahl < 4/nl oder > 12/nl

## Material und Methoden

Im Rahmen einer deutschlandweiten Umfrage zwischen Oktober 2019 und April 2020 zum Thema Antikoagulation und medikamentöser Thromboseprophylaxe bei Sepsis und Sepsis-induzierter Koagulopathie [16] wurde in einem gesonderten Fragenblock gezielt nach der verwendeten Sepsisdefinition und den entsprechenden Screeningwerkzeugen gefragt. Die Umfrage richtete sich ausschließlich an die *ärztlichen Leiter einer Intensivstation (ITS)* oder eines Intensivbereiches. Ziel war es, nur einen ausgefüllten Fragebogen pro Intensivbereich zu erhalten. Es könnten daher auch mehrere Intensivbereiche eines Klinikums geantwortet haben, sofern sie organisatorisch getrennt sind. Die Teilnahme erfolgte anonym, eine Mehrfachteilnahme war technisch nicht blockiert. Der Online-Fragebogen [16] wurde von der Arbeitsgruppe perioperative biomedizinische Informatik der Klinik für Anästhesiologe des Klinikums der Universität München mittels des Systems „Django-Survey“ aufgesetzt.

Die Details zu den Modalitäten der Umfrage sind bereits an anderer Stelle veröffentlicht, ebenso wie ein detaillierter Material- und Methodenteil [16]. Kernelement des Fragebogens, der auf den Umgang mit Sepsis-induzierter Koagulopathie (SIC) und vorbestehender Antikoagulation fokussierte, waren Fallvignetten zu pneumogener und abdomineller Sepsis. Daneben wurden Fragen zur „Infrastruktur“ und dem „Status quo“ bei der Behandlung einer Sepsis im Allgemeinen gestellt. Während Auswertungen der Fallvignetten an anderer Stelle publiziert sind [16], sollen die Antworten zum Fragenkomplex (3.) *„Status quo Sepsis“* hier gesondert vorgestellt werden [16]. In diesem Teil des Fragebogens konnten nur Einfachantworten gegeben werden. Dabei standen den Teilnehmern Drop-down-Menüs zur Verfügung. Wurde nach konkreten Zahlen gefragt (Anzahl der Intensivbetten, Liegedauer, durchschnittlicher Prozentsatz an beatmeten Patienten), so mussten konkrete Zahlenwerte eingegeben werden.

### Statistik

Es erfolgte eine deskriptive Auswertung mittels Microsoft® Office Excel (Excel für Mac Version 16.3, Microsoft Corporation, Redmond, WA, USA) sowie Prism® 8 for MacOS (GraphPad Prism® für Mac Version 8.3.0; GraphPad Software LLC, San Diego, CA, USA).

### Ergebnisse

**Figure 1:**
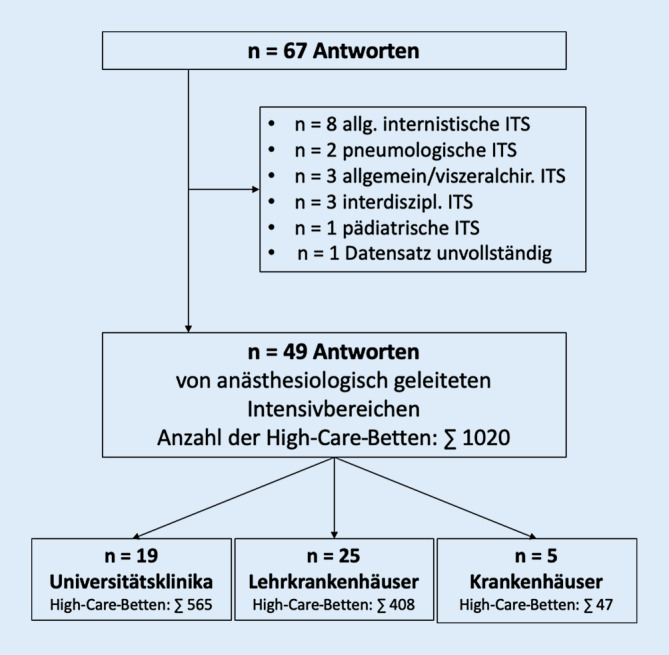

Insgesamt nahmen 67 Intensivstationen an der Befragung teil. In drei Viertel der Fälle (*n* = 50, 74,6 %) kamen die Antworten dabei von anästhesiologisch geleiteten Intensivstationen, während 10 Antworten von internistisch (allgemeine Innere Medizin und Pneumologie; 14,9 %), 3 von allgemein- und viszeralchirurgisch (4,5 %), 3 von interdisziplinär (4,5 %) und eine von pädiatrisch (1,5 %) geleiteten Intensivbereichen kamen [16]. Ein Datensatz wurde aufgrund großer Dokumentationslücken ausgeschlossen (Abb. 1). Knapp 39 % (*n* = 19) der Antworten von anästhesiologisch geleiteten Intensivbereichen kamen von Universitätsklinika, 51 % (*n* = 25) von akademischen Lehrkrankenhäusern und gute 10 % (*n* = 5) von kleineren Kliniken. Insgesamt führen die ausgewerteten Intensivstationen 1020 Intensivbetten, den größten Teil hiervon (55 %) an Universitätskliniken (Abb. 1). Die meisten der teilnehmenden Intensivstationen waren an Häusern verortet, die insgesamt > 1000 (*n* = 16), 501 bis 1000 (*n* = 14) oder 251 bis 500 (*n* = 15) Betten führen (Tab. 1). Jeweils zwei Drittel der teilnehmenden Intensivstationen an Universitätsklinika und akademischen Lehrkrankenhäusern gaben an, über 100 Fälle von Sepsis und septischem Schock pro Jahr zu behandeln (Tab. 1). Dabei haben nur knapp 75 % der teilnehmenden Universitätsklinika eine Standardarbeitsanweisung (SOP) für die Behandlung von Patienten mit Sepsis. Bei den Lehrkrankenhäusern sind es immerhin 80 % und bei den sonstigen Krankenhäusern 100 %.

**Table 1:** 

Bettenanzahl des Krankenhauses	Gesamt		Universitätsklinika		Lehrkrankenhäuser		Krankenhäuser	
–	*n*	%	*n*	%	*n*	%	*n*	%
< 100	1	2,0	0	0,0	0	0,0	0	0,0
100–250	3	6,0	0	0,0	0	0,0	3	60,0
251–500	15	30,0	0	0,0	13	52,0	2	40,0
501–1000	14	28,0	3	15,8	11	44,0	0	0,0
> 1000	16	32,0	15	78,9	1	4,0	0	0,0
K. A.	1	2,0	1	5,3	0	0,0	0	0,0
*Sepsisfälle pro Jahr*								
–	*n*	%	*n*	%	*n*	%	*n*	%
< 25	4	8,0	1	5,3	1	4,0	1	20,0
25–100	15	30,0	6	31,6	9	36,0	0	0,0
101–250	19	38,0	5	26,3	10	40,0	4	80,0
251–400	10	20,0	6	31,6	4	16,0	0	0,0
> 400	2	4,0	1	5,3	1	4,0	0	0,0
*SOP für Sepsistherapie vorhanden?*	*n*	*%*	*n*	*%*	*n*	*%*	*n*	*%*
Nein	10	20,4	5	26,3	5	20,0	0	0,0
Ja	39	79,6	14	73,7	20	80,0	5	100,0
*Mittlere Verweildauer*								
Mittelwert (Tage)	6,1	–	7,4	–	5,8	–	3,3	–
Median (Tage)	4,0	–	4,5	–	5,0	–	3,0	–
25.–75. Perzentile	4,0	6,8	4,0	7,0	4,0	7,0	3,0	4,0
*Durchschnittlicher Anteil an invasiv beatmeten Patienten*								
Mittelwert (Tage)	50,6	–	57,0	–	51,2	–	40,0	–
Median (Tage)	52,0	–	60,0	–	54,0	–	40,0	–
25.–75. Perzentile	31,5	70,0	28,5	80,0	38,8	67,0	29,8	45,0
*Anzahl der Intensivbetten (nur Beatmungsbetten, keine IMC)*								
Mittelwert (Tage)	21,2	–	31,4	–	17,0	–	9,4	–
Median (Tage)	16,0	–	14,5	–	14,5	–	6,0	–
25.–75. Perzentile	10,8	27,8	14,5	39,0	12,0	19,8	5,0	16,0
–	*n*	–	*n*	%	*n*	%	*n*	%
*Summe der Intensivbetten (nur Beatmungsbetten, keine IMC)*	1020	–	565	55,4	408	40,0	47	4,6

Gefragt wurde nach der durchschnittlich behandelten Zahl an Patienten mit Sepsis und septischem Schock. *IMC* intermediate Care, *SOP* Standardarbeitsanweisung (engl. Standard Operating Procedure), *K.* *A.* keine Angabe

**Figure 2:**
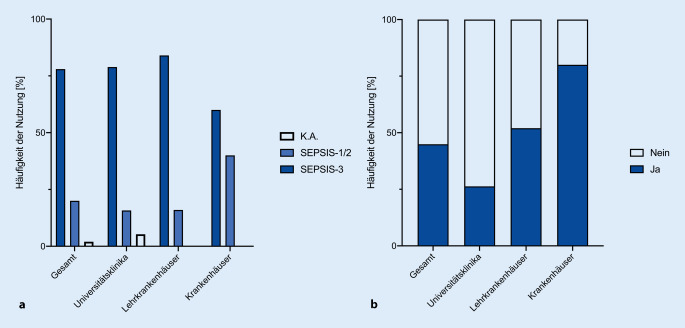

Die SEPSIS-3-Definition dient an 4 von 5 Universitätsklinika (78,9 %) und Lehrkrankenhäusern (84 %) als Arbeitsgrundlage zur Diagnose einer Sepsis (Abb. 2a). Der in der SEPSIS-3-Definition vorgeschlagene Screeningtest „qSOFA“ wird jedoch nur von 26,3 % der teilnehmenden „Universitätsklinika“, aber immerhin von 52 % der „Lehrkrankenhäuser“ und 80 % der „Krankenhäuser“ verwendet (Abb. 2b).

## Diskussion

Ziel der vorliegenden Umfrage war es, ein Bild darüber zu erhalten, inwieweit die SEPSIS-3-Definition 4 Jahre nach ihrer internationalen Einführung auf den deutschen Intensivstationen angekommen ist. Obwohl mit 49 nur ein kleiner Teil der anästhesiologisch geführten Intensivstationen in Deutschland an der Umfrage teilnahmen, erlaubt sie doch Rückschlüsse auf die allgemeine Akzeptanz der SEPSIS-3-Definitionen in der deutschen Anästhesiologie. So sind mit 19 von 35 immerhin über die Hälfte der deutschen Universitätskliniken (56 %) in unserer Umfrage vertreten [19]. Quantitativ weniger stark vertreten waren nichtuniversitäre Häuser: Hiervon gab es in Deutschland im Jahr 2018 *n* = 1101 mit Intensiv- und/oder Intermediate-Care-Stationen [19]. Von diesen waren 398 in öffentlicher Hand, 454 freigemeinnützige Krankenhäuser und 249 private Krankenhäuser. In wie vielen dieser Häuser eine anästhesiologisch geführte Intensivstation existiert, ist nicht bekannt. Trotzdem ist davon auszugehen, dass unsere Umfrage mit 30 Antworten nur einen sehr kleinen Teil dieser nichtuniversitären Häuser mit anästhesiologischen Intensivstationen erfasst. Die teilnehmenden Häuser waren zudem in erster Linie große Kliniken mit > 250 Betten. Insgesamt sind in unserer Arbeit damit Intensivstationen repräsentiert, die 14 % (1020 von 7230) der „High-Care-Betten“ verantworten, die im November 2020 im Register der Deutschen Interdisziplinären Vereinigung für Intensiv- und Notfallmedizin (DIVI) gemeldet waren [15]. Unsere Daten weisen einen deutlichen Bias hin zu großen universitären Häusern auf, sodass über das Vorgehen auf Intensivstationen an kleineren Häusern keine validen Aussagen getroffen werden können. Es kann jedoch angenommen werden, dass das Vorgehen an den erfassten großen Häusern über ihren Lehr- und Ausbildungsauftrag (und die Personalfluktuation) eine Strahlwirkung auf umliegende kleinere Häuser hat (untersucht und beschrieben in [8]) und der repräsentative Wert unserer Umfrage daher höher ist, als die reinen Zahlen vermuten lassen. Unserer Ergebnisse zeigen, dass die SEPSIS-3-Definition inzwischen zumindest in den anästhesiologisch geführten Intensivstationen der Universitätsklinika in Deutschland angekommen ist und SEPSIS-1/2 weitgehend abgelöst hat. Dies ist eine deutliche Veränderung im Vergleich zum Jahr 2018, als in einer Umfrage auf Intensivstation noch über 50 % der Teilnehmenden angaben, alle 3 Definitionen parallel zu verwenden [11]. Hierbei ist anzumerken, dass die Verwendung der SEPSIS-3-Definition zumindest seit der Veröffentlichung der ICD-10 Version 2020 [6] hilft, Sepsis korrekt zu verschlüsseln. Denn in dieser neusten Ausgabe des ICD-10 sind die Codes für SIRS und SIRS mit Organkomplikation zwar noch vorhanden, aber de facto wertlos, da der Nutzer angehalten wird, zunächst stets die „die Sepsis auslösende Grunderkrankung“ (z. B. A41.–) zu kodieren. Die Codes für Sepsis (R65.0!) und schwere Sepsis (R65.1!) sind heute nicht mehr als Hauptdiagnose zur Abrechnung zulässig [2, 6]. Stattdessen soll zunächst, wie von der DSG vorgeschlagen [5], z. B. A41.9 (Sepsis, nicht näher bezeichnet) verschlüsselt werden und sekundär ein Code für eine oder mehrere Organdysfunktionen als Nebendiagnosen vergeben werden (z. B. R57.2 septischer Schock, N17.– akutes Nierenversagen, J96.– akute respiratorische Insuffizienz, D65.1 disseminierte intravasale Koagulopathie, G93.4 Enzephalopathie – nicht näher bezeichnet oder K72.0 akutes oder subakutes Leberversagen) [5]. Die Erfassung der Codes für Organversagen (oder Sepsis, schwere Sepsis) als Nebendiagnosen erhöht dabei jedoch nicht die effektive Bewertungsrelation der Diagnosis Related Group (DRG) und führt damit nicht zu einem höheren Erlös. Für die Abrechnung in der Klinik ist es daher zunächst egal, ob eine Sepsis, ein septischer Schock (mit oder ohne weitere Organversagen) oder eine Sepsis mit SIRS kodiert wird. Erst die Dokumentation weiterer spezifischer Verfahren (z. B. Beatmungsstunden) führt zu einer höheren effektiven Bewertungsrelation der DRG. Für die Klinik birgt dies die Gefahr, dass eine nachlässige Kodierung potenziell zu Rückforderungen durch den medizinischen Dienst der Krankenkassen (MDK) führen kann.

Ein weiterer interessanter Aspekt unserer Untersuchung sind unsere Ergebnisse zur Verwendung des Testinstruments „qSOFA“ für die Identifikation von Sepsisverdachtsfällen außerhalb von Intensivstationen. Hier zeigen unsere Daten, dass Universitätsklinika bezüglich der Verwendung des qSOFAs deutlich zurückhaltender sind als andere Häuser und den qSOFA häufig nicht verwenden, obwohl SEPSIS‑3 bereits eingeführt ist. Dies könnte darin begründet sein, dass der qSOFA-Score zwar in der SEPSIS-3-Definition als Screeninginstrument vorgeschlagen wird, in nachfolgenden Untersuchungen jedoch gezeigt werden konnte, dass er zwar sehr spezifisch Patienten mit Organversagen identifiziert, in Bezug auf seine Sensitivität aber anderen Screeninginstrumenten wie den SIRS-Kriterien oder dem NHS-early Warning Score (NEWS) deutlich unterlegen ist [3, 9, 12, 14, 20]. Stattdessen identifiziert der qSOFA-Score Patienten, deren Letalität mit 23 % deutlich über der einer einfachen Sepsis (etwa 10 %) liegt [7, 18]. Dies ist insofern kritisch, als dass ein Testinstrument, das im Rettungsdienst, in der Notaufnahme oder auf Normalstationen eingesetzt wird, im Sinne der Patientensicherheit über eine hohe Sensitivität verfügen sollte. Damit kann nichtärztliches medizinisches Personal Patienten identifizieren, die einer genaueren ärztlichen Untersuchung unterzogen werden müssen, welche spezifischere Testmetoden enthalten kann. Einen spezifischen Test einzusetzen, der lediglich eine Sensitivität von 30–70 % aufweist [9, 12, 20], kann hingegen eine falsche Sicherheit suggerieren und zu einer verspäteten Erkennung von Sepsisverdachtsfällen und damit zu einer erhöhten Letalität führen [17]. Die Leitlinie der DSG berücksichtigt die Besonderheiten des qSOFA-Scores insofern, als dass (lediglich) empfohlen wird, ihn „regelmäßig zu bestimmen, um Risikopatienten mit vitaler Bedrohung frühzeitig zu erkennen“ [4]. In der Begründung dieser Empfehlung wird auf die begrenzte Evidenz bezüglich des Stellenwertes des qSOFA-Scores bei der Diagnose der Sepsis eingegangen [4]. Der qSOFA ist dabei der einzige Score, der in der Leitlinie explizit genannt wird, sodass der Eindruck entstehen kann, mit der ebenfalls in der Leitlinie enthaltenen allgemeinen Empfehlung zur „Implementierung […] eines Screenings für Risikopatienten“ sei ausschließlich der qSOFA-Score gemeint. Ob an den Kliniken, die den qSOFA-Score zum Screening auf Normalstationen einsetzen, darüber hinaus noch ein weiterer Score verwendet wird, wird durch unsere Daten nicht abgebildet. Eine Spezifizierung der Empfehlungen wäre jedoch sinnvoll, um eine frühzeitige Erkennung von Patienten mit Sepsis, die zwar (im Sinne der Sepsisdefinition) lebensbedrohlich erkrankt, aber (noch) nicht per-akut gefährdet sind, sicher zu gewährleisten. Nur so kann effektiv verhindert werden, dass die Leitlinie zur Abschaffung von sensitiveren Screeninginstrumenten und damit potenziell zu einer Unterversorgung von Sepsispatienten beiträgt.

Zusammenfassend kann konstatiert werden, dass der Übergang von der SEPSIS-2- auf die SEPSIS-3-Definition in Deutschland weit vorangeschritten ist und diese bereits gelebte Realität in Bezug auf die Definition der Sepsis durch die Einführung des ICD-11 nun bald auch formal abgebildet werden kann. Unsere Daten zeigen, dass auch der qSOFA-Score bereits Teil des Krankenhausalltags in Deutschland geworden ist. Seine Schwächen in Bezug auf die Sensitivität implizieren jedoch die Notwendigkeit einer Diskussion darüber, ob er als alleiniges Sepsistestinstrument im Rettungs- und Notarztdienst, in Notaufnahmen und auf Normalstationen zum Einsatz kommen sollte. Eine entsprechende interdisziplinäre Fachgesellschafts-übergreifende Empfehlung wäre daher wünschenswert.
